# Novel Mutant AAV2 Rep Proteins Support AAV2 Replication without Blocking HSV-1 Helpervirus Replication

**DOI:** 10.1371/journal.pone.0170908

**Published:** 2017-01-26

**Authors:** Michael Seyffert, Daniel L. Glauser, Elisabeth M. Schraner, Anna-Paula de Oliveira, Jorge Mansilla-Soto, Bernd Vogt, Hildegard Büning, R. Michael Linden, Mathias Ackermann, Cornel Fraefel

**Affiliations:** 1 Institute of Virology, University of Zurich, Zurich, Switzerland; 2 Whitehead Institute for Biomedical Research, Cambridge, Massachusetts, United States of America; 3 Institute of Veterinary Anatomy, University of Zurich, Zurich, Switzerland; 4 Center for Cell Engineering, Memorial Sloan Kettering Cancer Center, New York, New York, United States of America; 5 Center for Molecular Medicine Cologne, University of Cologne, Cologne, Germany; 6 Institute for Experimental Hematology, Hannover Medical School, Hannover, Germany; 7 Department of Infectious Diseases, King’s College London, London, United Kingdom; National Institute of Dental and Craniofacial Research, UNITED STATES

## Abstract

As their names imply, parvoviruses of the genus *Dependovirus* rely for their efficient replication on the concurrent presence of a helpervirus, such as herpesvirus, adenovirus, or papilloma virus. Adeno-associated virus 2 (AAV2) is such an example, which in turn can efficiently inhibit the replication of each helpervirus by distinct mechanisms. In a previous study we have shown that expression of the AAV2 *rep* gene is not compatible with efficient replication of herpes simplex virus 1 (HSV-1). In particular, the combined DNA-binding and ATPase/helicase activities of the Rep68/78 proteins have been shown to exert opposite effects on the replication of AAV2 and HSV-1. While essential for AAV2 DNA replication these protein activities account for the Rep-mediated inhibition of HSV-1 replication. Here, we describe a novel Rep mutant (Rep-D371Y), which displayed an unexpected phenotype. Rep-D371Y did not block HSV-1 replication, but still supported efficient AAV2 replication, at least when a double-stranded AAV2 genome template was used. We also found that the capacity of Rep-D371Y to induce apoptosis and a Rep-specific DNA damage response was significantly reduced compared to wild-type Rep. These findings suggest that AAV2 Rep-helicase subdomains exert diverging activities, which contribute to distinct steps of the AAV2 life cycle. More important, the novel AAV2 mutant Rep-D371Y may allow deciphering yet unsolved activities of the AAV2 Rep proteins such as DNA second-strand synthesis, genomic integration or packaging, which all involve the Rep-helicase activity.

## Introduction

**Fig 1:**
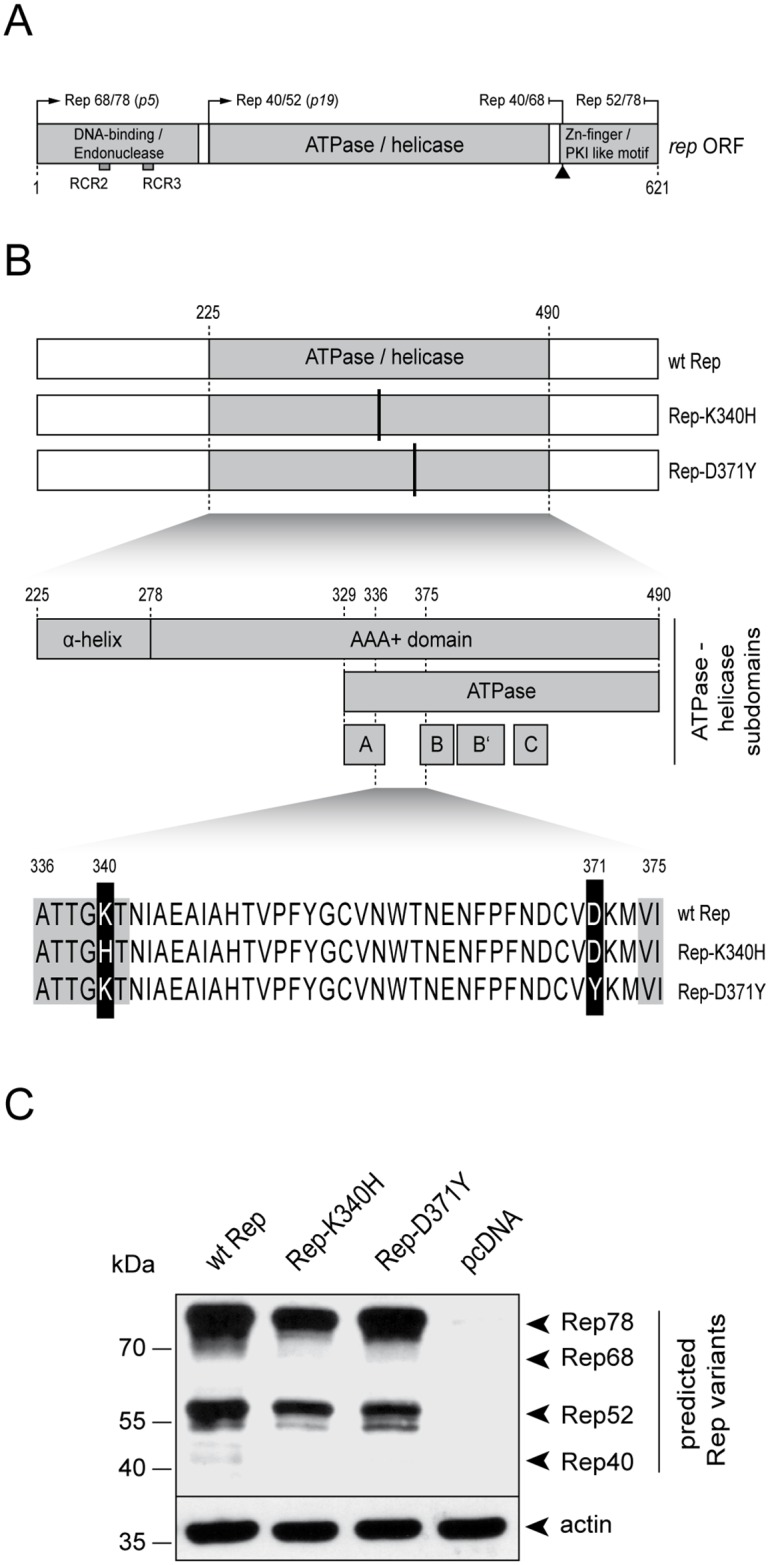
The AAV2 Rep protein domains and the Rep expression plasmids analyzed in this study. (A) Schematic representation of the main protein domains within the rep ORF (grey bars). The promoters p5 and p19 responsible for the expression of the different Rep proteins are indicated. The common splicing site at the C-terminal end of the ORF is depicted with an up-facing arrow head. (B) Schematic representation of the AAV2 Rep proteins analyzed in this study; (i) the wild-type (wt) Rep, (ii) the helicase-deficient mutant Rep-K340H and (iii) the helicase mutant Rep-D371Y. The ATPase/helicase domain (grey box) and the corresponding mutations (vertical black lines) are indicated. A detailed representation of the helicase domain (aa 225–490) is shown below. The mutation K340H is located within the Walker motif A whereas the mutation D371Y is located between the Walker motifs A and B. The corresponding locations within the Rep aa-sequence are indicated. (C) Rep protein levels. Plasmids encoding either the wt Rep, the mutant Rep-K340H, the mutant Rep-D371Y or no Rep (pcDNA) were transfected into Vero cells. At 24 hrs after transfection, the cells were harvested and processed for Western analysis using a Rep-specific antibody. The different Rep protein variants are indicated on the right. Detection of actin served as a loading control.

Adeno-associated virus 2 (AAV2) is a helper virus-dependent human parvovirus with a unique biphasic life cycle. In presence of a helpervirus such as herpes simplex virus 1 (HSV-1), adenovirus 2 (AdV2), or human papillomavirus 16 (HPV-16), it undergoes lytic replication [1–4], while in absence of a helpervirus, it establishes latency. The AAV2 particle consists of a small icosahedral capsid enclosing a single-stranded (ss)DNA genome of approximately 4,700 nucleotides [5]. The AAV2 genome contains inverted terminal repeats (ITRs) at both ends flanking two clusters of genes, *rep* and *cap*, which due to splicing events, alternative start codons, and nested open reading frames (ORF) encode a total of eight proteins from three different promoters. [6–9]. The ITRs form hairpin structures and contain a Rep-binding site (RBS) as well as a terminal resolution site (trs), which together act as viral origin of DNA replication [10, 11]. Among the eight proteins encoded by AAV2 are four different Rep proteins termed Rep40, Rep52, Rep68, and Rep78, which differ in their apparent molecular weight as well as in the composition of their structural and functional domains [12] (Fig 1A). At the very N-terminal region of the *rep* open-reading frame (ORF) the combined DNA-binding and endonuclease domains are located [13–15] (Fig 1A). The DNA-binding domain (map position 1–200) is responsible for binding to double-stranded (ds)DNA templates at specific Rep binding site (RBS) motifs consisting of the minimal consensus sequence GAGYGAGC [16], which are located within the AAV2 ITRs and the p5 promoter region. The DNA-binding domain harbors two rolling circle replication (RCR) motifs termed RCR2 and RCR3 [14, 17], which comprise the endonuclease activity which is essential for the terminal resolution process during DNA replication [17], as well as for integration of the AAV2 genome into the host cell genome to establish latency [18–20] (Fig 1A). In the center of the *rep* ORF the Rep-helicase is located (map position 225–490) (Fig 1A and 1B). This particular viral helicase belongs to the superfamily 3 (SF3) helicases, which are encoded mainly by small DNA viruses [21]. The complete helicase domain can be subdivided into two main components, the α-helix domain and the AAA+ region (Fig 1B). The α-helix domain is located at map position 225 to 278 and is responsible for hexamerization. The AAA+ motif is located at map position 278 to 490 [22, 23] and can be further divided into several sub-domains, including the ATPase domain (map position 329–490) [23], which contains four Walker motifs [22]. The core of the SF3 ATPase domain constitutes the Walker motifs A, B and B’, which are highly conserved entities also found in SF1 and SF2 helicases [21]. Unlike other helicases, the SF3 helicase contains a third type of Walker motif, C, which is located between B’ and the rest of the C-terminal protein domain [24] (Fig 1B). The Walker motifs A, B and B’ represent residues that directly interact with Mg-ATP as well as Mg-ADP, respectively, and are critical for NTP stability during NTP turnover [21, 24–27]. The Walker motif C however, is bearing a polar residue, which is mediating interactions necessary for NTP hydrolysis rather than NTP binding [21, 28]. Typically, SF3 helicases form hexameric rings and have been shown to either specifically bind sites on dsDNA templates via the N-terminal DNA-binding domain, or non-specifically bind ssDNA templates via the helicase domain itself [26, 29, 30]. The combined ATPase/helicase domain is required for AAV2 DNA replication and packaging of the ssDNA genome into pre-assembled capsids [31, 32]. At the C-terminal end of the *rep* ORF a protein kinase A (PKA) binding site is located (map position 526–621) and consists of a Zn-finger motif [33, 34] and a PKA inhibitor (PKI)-like motif [35] (Fig 1A). While the Zn-finger motif is responsible for inhibition of cell cycle progression [34, 36], the PKI-like motif is responsible for inhibition of AdV DNA replication [35]. The smaller Rep40/52 proteins, which originate from transcripts controlled by the p19 promoter, and the larger Rep68/78 proteins which originate from transcripts controlled by the p5 promoter, all comprise the ATPase/helicase domain. However, the DNA binding and endonuclease domains are part of the Rep68/78 proteins only and the C-terminal Zn-finger and PKI-like motifs are part of the unspliced Rep52 and Rep78 proteins only (Fig 1A).

AAV2 has developed distinct strategies to inhibit helpervirus replication, likely to reduce competitive interactions within the co-infected cell [37–43]. In previous studies, we have demonstrated that the expression of the AAV2 Rep68/78 proteins leads to a significant inhibition of HSV-1 DNA replication [37, 38]. Moreover, we found that the AAV2 Rep protein domains responsible for the inhibition of HSV-1 DNA replication include the DNA-binding and the ATPase/helicase activities, while the endonuclease activity is not required [38, 44]. In those studies, we have utilized a set of mutant Rep proteins lacking distinct activities or domains to assess their effect on the production of infectious HSV-1 particles. We demonstrated that Rep68, Rep78 and Rep68/78-Y156F, which has a mutation within the RCR2 motif required for endonuclease activity [17, 45, 46], reduce titers of infectious HSV-1 particle stocks by approximately 100-fold, whereas Rep52, which lacks the DNA binding domain, and Rep68/78-K340H, which is deficient for helicase activity [47], do not [38]. Of note, the mutant Rep-K340H was generated by introducing a two-base change within the Lysine (K) codon at position 340. In particular, the codon AAG (K) was converted to CAC (H) (Fig 1B) [47, 48]. This mutation is located within the Walker motif A and directly affects the purine binding capacity and therefore completely abrogates helicase activity. In conclusion, those results suggested that the Rep activities required for AAV2 replication precisely coincide with the activities responsible for inhibition of HSV-1 replication and that efficient HSV-1 replication is not compatible with expression of a functional AAV2 *rep68/78* gene.

Here, we describe the phenotype of a new Rep mutant (D371Y) which harbors a Tyrosine (Y) in place of an Aspartic-acid (D) at position 371 of the amino acid sequence. This mutant was obtained by a single-base substitution (G→T) generating a TAC in place of a GAC codon and is located between Walker motifs A and B (Fig 1B). This Rep mutant displayed an unexpected phenotype, as it did not block the replication of HSV-1 but was still capable of supporting AAV2 replication, at least when dsAAV2 genomes were used as template. In addition, we found that the capacity of Rep-D371Y to induce apoptosis and DNA damage responses in transfected cells was significantly reduced compared to wild-type (wt) Rep.

## Materials and Methods

### Cells and viruses

Vero (ATCC—LGC Standards GmbH, Wesel, Germany) and Vero 2–2 cells [49] were maintained in Dulbecco’s modified Eagle medium supplemented with 10% fetal bovine serum, 100 units/ml penicillin G, 100 μg/ml streptomycin, and 0.25 μg/ml amphotericin B. For culturing Vero 2–2 cells, 500 μg/ml G418 was added to the medium. All cell cultures were kept at 37°C and 5% CO_2_. The wt HSV-1 (strain F) was grown in Vero cells and titers were determined as described previously [38, 44]: briefly, confluent monolayers of Vero cells were infected with wt HSV-1 at a MOI of 0.1 and incubated until the cytopathic effect (CPE) reached 100%. Then, the cells were lysed by three freeze/thawing-cycles and cellular debris was separated from the virus stock by centrifugation for 10 min at 1,900 x *g*. The cleared lysate was titrated on Vero cells using the Spearman-Karber method. Virus stocks were kept at -80°C for long term storage. Production of wt AAV2 and rAAV2-D371Y stocks, followed by purification on a Iodixanol gradient was performed as described elsewhere [50–52]. Transfection of Vero and Vero 2–2 cells was performed using the Lipofectamine^™^ LTX and Plus^™^ reagents from Invitrogen^™^ (Thermo Fisher Scientific, Reinach, Switzerland) according to the manufacturers’ protocol.

### Plasmids

The plasmids expressing the wt Rep (pcDNA.Rep68/78) and the mutant Rep-K340H (pcDNA.Rep68/78-K340H) genes were described elsewhere [38]. The plasmid expressing the mutant Rep-D371Y was cloned by PCR. A first round of PCR was performed using the pcDNA.Rep68/78 plasmid DNA as a template with primers reaching from the first *Bam*HI-site (nt1040; relative to the AAV2 genome) to the *Acc*I-site (nt1420; relative to the AAV2 genome) where in the reverse primer the G→T substitution at position 1431 was introduced (for. primer: GCAGTGGATCCAGGAGGACCAGGCCTCATA / rev. primer: ACCAGATCACCATCTTGTAGACACAGTCGT). A second round of PCR was performed with primers reaching from *Acc*I-site (nt1413) to the *Xho*I-site (nt2225) (for. primer: CCCTTCAACGACTGTGTCTACAAGATGGTG / rev. primer: CTTCAGAGAGAGTGTCCTCGAGCCAATCTG). The two PCR fragments were ligated between the *Bam*HI and *Xho*I sites of pcDNA.Rep68/78 plasmid DNA. The empty plasmid backbone pcDNA3.1+ was purchased from Invitrogen (Thermo Fisher Scientific, Reinach, Switzerland, Cat.no. V790-20). The mRFP expressing plasmid (pcDNAmRFP) was obtained from Michele Gastaldelli (University of Zurich). The amplicon vectors pHSV-TetO [37] and pAV2-LacO [53] were described previously. The plasmid pSV2-eYFP/lacI, expressing enhanced yellow fluorescent protein (eYFP) linked to the *lac* repressor protein (LacI) [54], was kindly provided by D. L. Spector (Cold Spring Harbor Laboratory, Cold Spring Harbor, NY). Plasmid pEYFPTetR expressing eYFP linked to the *tet* repressor protein (TetR) was described elsewhere [55, 56]. pHSVGFP, an HSV-1 amplicon vector containing the GFP-coding sequence under the control of the HSV-1 IE 4/5 promoter [57] and pAV2GFP, a recombinant AAV (rAAV) plasmid containing the GFP-coding sequence under the control of the HCMV IE1 enhancer/promoter flanked by the AAV2 ITRs [53], were described elsewhere. The BAC-cloned HSV-1 genome deficient for the packaging signal *pac* and the gene *icp27* (fHSVΔpacΔ27Δkn) together with pEBHICP27 represent a replication-competent, packaging-defective HSV-1 genome and were described previously [58]. The hybrid vector plasmid pHyRaNGFPa was described in [53]. In brief, the plasmid pRep harboring the full-length Rep ORF was cut with NotI and the overhang-ends were blunt-end repaired with T4 DNA polymerase. This 2.5-kb fragment was inserted into the blunt-ended SphI site of pHSVNot resulting in pHyRa. In parallel, the BglII fragment was excised from the pAV2GFP plasmid which contains the ITR-flanked GFP cassette and was inserted into the BamHI site of pUC18-Not. The resulting plasmid pAV2GFP-Not was cleaved with NotI and the 2.3-kb fragment containing the ITR-flanked transgene gfp was inserted into the unique NotI site on pHyRa, forming pHyRaNGFPa. To generate the plasmid pHyRD371YGFPa, the same strategy was used to generate pHyRaNGFPa, but instead a pRep plasmid was used harboring the mutation D371Y (pRep-D371Y). pRep-D371Y was cloned by ligating the PstI fragment from the pcDNA.Rep78-D371Y template directly into the PstI cleaved target plasmid pRep. pcDNA.eGFP expressing eGFP under the control of the HCMV IE1 enhancer/promoter was described previously [38]. The plasmid pAV2-D371Y has been constructed as follows: the 837 bp BamHI (partial digestion)-HindIII fragment of pAV2 was replaced by the 837 bp BamHI-HindIII fragment of pcDNA.Rep-D371Y.

### Rep protein synthesis and purification

The wt Rep68 and the mutant Rep68-D371Y proteins were expressed and purified as described elsewhere [26, 59].

### Antibodies

#### Primary antibodies

The mouse anti-AAV Rep mAb (clone 303.9) was purchased from Fitzgerald Industries International (Acton, MA, USA), the mouse anti-Actin mAb (clone AC-47) from Sigma (Sigma-Aldrich Chemie GmbH Buchs, Switzerland), the mouse anti-phospho-ATM (S1981) mAb (clone 10H11.E12) from Rockland Immunochemicals Inc. (Limerick, PA, USA), the rabbit anti-phospho-RPA32 (S4/S8) pAb BL- A300-245A from Bethyl Laboratories (Montgomery, TX, USA), the mouse anti-phospho-H2A.X (S139) mAb (clone JBW301) from Upstate Millipore (Merck AG, Zug, Switzerland) and the mouse anti-GFP MAb JL-8 from Clontech (Takara Bio Europe SAS). The alkaline phosphatase-conjugated anti-DIG antibody (Anti-Digoxigenin-AP, Fab fragments, 11093274910) was purchased from Roche Applied Science (Roche Diagnostics, Rotkreuz, Switzerland).

#### Secondary antibodies

Goat anti-mouse IgG(H+L)-Alexa Fluor 488 (AF488), goat anti-mouse IgG(H+L)-AF594 and goat anti-rabbit IgG(H+L)-AF488 were purchased from Molecular Probes (Thermo Fisher Scientific, Reinach, Switzerland). Rabbit anti-mouse IgG (whole molecule)-peroxidase was purchased from Sigma (Sigma-Aldrich Chemie GmbH Buchs, Switzerland).

### Western blot analysis

Vero cells were transfected with 0.5 μg of the individual pcDNA.Rep plasmids or empty pcDNA3.1+ vector. At 24 h after transfection, the cells were lysed and processed for Western analysis as described previously [37]. Primary antibodies were used at the following dilutions: mouse anti-AAV Rep mAb clone 303.9, 1:100; mouse anti-GFP MAb JL-8 1:8,000, rabbit anti-mouse IgG (whole molecule)-peroxidase, 1:10,000.

### Immunofluorescence analysis

Immunofluorescence staining was performed as described previously [37]. The primary antibodies were used at the following dilutions: mouse anti-AAV Rep MAb, 1:100; rabbit anti-phospho-RPA32 (S4/S8) pAb, 1:100; mouse anti-phospho-ATM (S1981) mAb, 1:100; and mouse anti-phospho-H2AX (S139) mAb, 1:100. Alexa Fluor-conjugated secondary antibodies were diluted 1:500. Confocal laser scanning microscopy (CLSM) was performed with a SP2 confocal microscope from Leica (Leica Microsystems, Heerbrugg, Switzerland) as described previously [60].

### HSV-1 amplicon packaging assay

Packaging of the HSV-1 amplicons pHSVGFP was performed as described previously [38, 58]. Briefly, Vero 2–2 cells were transfected with 0.5 μg of pHSVGFP, 2 μg of fHSVΔpacΔ27Δkn, and 0.2 μg of pEBHICP27 together with the indicated amounts of pcDNA.Rep plasmids or empty pcDNA3.1+ vector. Three days later, cells were harvested and HSV-1 amplicon particles were purified as described above for wt HSV-1 stocks. The purified HSV-1 amplicon stocks were titrated by infection of Vero cells and enumeration of EGFP+ cells 48 hpi by flow cytometry on a Gallios Flow Cytometer (Beckman Coulter International SA, Nyon, Switzerland) and analyzed with the Kaluza Analysis-Software (Beckman Coulter International SA, Nyon, Switzerland).

### Southern analysis

Southern analysis was performed essentially as described previously [38]. Briefly, Vero 2–2 cells were co-transfected with the different Rep encoding plasmids (pcDNA.Rep) or the empty backbone plasmid pcDNA3.1+ together with the AAV2 replicon plasmid pAV2GFP and infected with wtHSV-1 (MOI 2) one day later. Three days after infection, the cells were harvested and extrachromosomal DNA was extracted [61]. The DNA was digested with DpnI to cut bacterial input DNA, separated on a 1% agarose gel, and transferred to a positively charged nylon membrane (Hybond N+ from Amersham—GE Healthcare, Opfikon, Switzerland). Hybridization with a digoxigenin (DIG)-labeled probe specific for the GFP-coding sequence and immunological detection using an alkaline phosphatase-conjugated anti-DIG antibody and chemiluminescence substrate (CDP Star) were performed as described by the supplier (Roche Applied Science, Rotkreuz, Switzerland). The DIG-labeled probe was produced by PCR amplification as described previously [38].

### Annexin V staining

Apoptosis assays were performed as described elsewhere [38]. Briefly, Vero cells were transfected with 0.25 μg pEGFP-N3 together with 0.5 μg pcDNA.Rep68/78 plasmids or empty pcDNA3.1+ vector as indicated. Annexin V staining was performed using the annexin V-Cy5 Apoptosis Detection Kit from Abcam (Lucerna-Chem AG, Luzern, Switzerland) according to the manufacturer’s protocol. The cells were analyzed by flow cytometry using a Gallios Flow Cytometer (Beckman Coulter International SA, Nyon, Switzerland) with filters specific for eGFP (transfected cells) and Cy5 (annexin V+ cells) and analyzed with the Kaluza Analysis-Software (Beckman Coulter International SA, Nyon, Switzerland).

### Electron microscopy

Virus preparations were adsorbed to carbon coated parlodion films mounted on 300 mesh/inch copper grids (EMS, Fort Washington, PA, USA) for 10 min, washed once with distilled water, and stained with saturated uranylacetate (Fluka, Buchs, Switzerland) for 1 min at room temperature. Specimens were analyzed in a transmission electron microscope (CM 12, Philips, Eindhoven, The Netherland) equipped with a CCD camera (Ultrascan 1000, Gatan, Pleasanton, CA, USA) at an acceleration voltage of 100 kV.SV-1.

### Gel filtration chromatography

Binding capacity of wt Rep68 and mutant Rep68-D371Y to either ssDNA or dsDNA (RBS) was performed as described elsewhere [59]. Briefly, wt Rep68 or mutant Rep68-D371Y proteins (16.6 μM) were incubated in the absence or presence of 2.8 μM ssDNA (polydT_25_) or 2.8 μM RBS dsDNA (28-mer: generated with the oligos 5’ GCCTCAGTGAGCGAGCGAGCGCGCAGAG 3’ and 5’ CTCTGCGCGCTCGCTCGCTCACTGAGGC 3’) for 30 min on ice. Samples (50 mL) were chromatographed on a Superose 6 10/300 GL column (GE Healthcare) with a flow rate of 0.5 ml/ min. Protein elution was detected by UV-light at 280 nm.

## Results

### Expression levels of the wild-type and mutant *rep* genes

In order to characterize the phenotype of the Rep mutant D371Y, we compared three different Rep constructs; (i) the wt Rep, (ii) the helicase-null mutant Rep-K340H, and (iii) the mutant Rep-D371Y (Fig 1B). All *rep* genes were cloned into the expression vector pcDNA3.1+ and are under the control of the constitutively active CMV IE1 enhancer/promoter. To estimate the expression levels, Vero cells were transfected with the individual Rep encoding plasmids and subjected to Western analysis 24 hrs later using a Rep-specific antibody. Actin staining served as a loading control. As shown in Fig 1C, the accumulation levels of the different Rep protein variants encoded by the respective expression plasmids were comparable.

### The Rep mutants Rep-D371Y and Rep-K340H both allow HSV-1 DNA replication, whereas wt Rep does not

**Fig 2:**
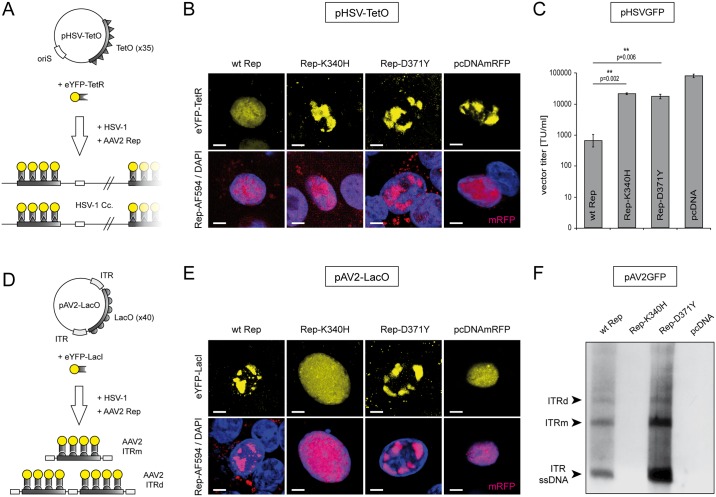
Effects of the different Rep constructs on HSV-1 and AAV2 DNA replication. (A) Replication of an HSV-1 replicon is visualized with plasmid pHSV-TetO containing an HSV-1 origin of DNA replication (oriS) and a TetO-cassette consisting of 35x TetR binding sites. In presence of HSV-1 replication factors (HSV-1), the accumulation of pHSV-TetO replication products is visualized by binding of an eYFP-TetR fusion protein (yellow fluorescent RCs). (B) Cells expressing the different Rep constructs (indicated on top) were analyzed for the ability to inhibit HSV-1 replication. The presence of Rep proteins was confirmed by staining with a Rep-specific antibody (red; bottom panels). A cell transfected with pcDNAmRFP expressing the monomeric red fluorescent protein (mRFP) was used as a positive control. DAPI was used to stain the nuclei. Scale bar; 5μm. (C) Effects of the different Rep constructs on HSV-1 amplicon vector production. Vero 2–2 cells were transfected with the HSV-1 amplicon DNA (pHSVGFP), packaging-defective HSV-1 helper DNA (fHSVΔpacΔ27Δkn), HSV-1 ICP27 encoding plasmid (pEBHICP27) and the different Rep encoding plasmids. At 72 hrs post transfection the pHSVGFP amplicon vector particles were harvested and titrated on Vero cells. The data are shown as means ± standard errors (SE) from three independent experiments. Asterisks indicate statistically significant differences based on a paired two-tail Student t-test (** = p<0.01). (D) Replication of an AAV2 replicon is visualized with plasmid pAV2-LacO, which is harboring a LacO-cassette consisting of 40x LacI binding sites flanked by AAV2 ITRs. In presence of the different Rep constructs and HSV-1 helper factors (HSV-1), the accumulation of pAV2-LacO replication products is visualized by binding of an eYFP-LacI fusion protein (yellow fluorescent RCs, top panels). (E) Cells expressing the different Rep constructs (indicated on top) were analyzed for the ability to replicate pAV2-LacO and accumulate corresponding RCs. A Rep-specific antibody was used to confirm the synthesis of Rep proteins in the transfected cells (red; bottom panels). A cell expressing mRFP was used as a negative control. DAPI was used to stain the nuclei. Scale bar; 5μm. (F) Vero 2–2 cells were transfected with pAV2GFP and the different Rep constructs as indicated. At 24 hrs after transfection, the cells were infected with HSV-1 (MOI 2) and subjected to Hirt DNA extraction 48hrs later. Extrachromosomal DNA was digested with DpnI and analyzed by Southern blotting with a DIG-labeled probe specific for GFP. The ITR ssDNA, the monomeric (ITRm) and the dimeric (ITRd) AAV2 replication intermediates are indicated on the left.

To assess the effects of the different Rep constructs on HSV-1 DNA replication we used a previously described visualization system employing the interaction of the tetracycline operator (TetO) located within a HSV-1 replicon plasmid (pHSV-TetO) with the tetracycline repressor (TetR) DNA-binding domain fused to the enhanced yellow-fluorescent protein (eYFP-TetR) (Fig 2A) [37, 56, 62]. Vero cells were co-transfected with pHSV-TetO, the eYFP-TetR expressing plasmid (pSV2eYFP-TetR) and the individual *rep* expression plasmids. The following day, the cells were infected with wt HSV-1 at a multiplicity of infection (MOI) of 2 and, 24 hrs later, fixed and stained with a Rep-specific antibody. Then, the formation of pHSV-TetO replication compartments (RCs) in Rep-positive cells was visualized by confocal laser scanning microscopy (CLSM). As shown in Fig 2B, the Rep mutants Rep-K340H and Rep-D371Y both allowed the formation of mature pHSV-TetO RCs while specifically in cells expressing the wt Rep proteins, pHSV-TetO RCs were not observed. A cell transfected with a control plasmid expressing the monomeric red fluorescent protein (mRFP), which allowed the formation of a mature HSV-1 RC was used as a positive control.

An HSV-1 packaging assay was utilized to quantify the effects of the different Rep constructs on the production of HSV-1 particles [38]. Briefly, Vero 2–2 cells were co-transfected with the individual *rep* expression plasmids, HSV-1 helper DNA which provides HSV-1 replication and packaging factors [58], and the HSV-1 amplicon plasmid pHSVGFP, which contains an HSV-1 origin of DNA replication, an HSV-1 DNA packaging/cleavage signal, and a GFP transgene cassette [53, 63, 64]. Three days after transfection, the pHSVGFP amplicon vector particles were harvested and titrated on Vero cells. Compared to the positive control (wt Rep) both Rep mutants (K340H and D371Y) significantly rescued pHSVGFP titers by more than 10-fold and at comparable levels (Fig 2C). The helicase-null mutant Rep-K340H, which cannot support AAV2 replication, was previously known to allow HSV-1 replication [38, 44]. As the mutant Rep-D371Y allowed HSV-1 replication, we expected that it may not support AAV2 DNA replication either. However, the following experiment revealed that Rep-D371Y has the unexpected property that it can support AAV2 DNA replication without blocking HSV-1 DNA replication.

### Unlike the mutant Rep-K340H, the mutant Rep-D371Y supports AAV2 DNA replication

To test the capability of the different Rep constructs to support AAV2 DNA replication we used a previously described visualization system employing the interaction of the Lac operator (LacO) located within an AAV2 replicon plasmid (pAV2-LacO) with the Lac repressor (LacI) DNA-binding domain fused to eYFP (eYFP-LacI) (Fig 2D) [55]. Vero cells were co-transfected with pAV2-LacO, the eYFP-LacI expressing plasmid (pSV2eYFP-LacI), and the individual *rep* expression plasmids. The following day, the cells were infected with wt HSV-1 (MOI of 2) and, 24 hrs later, fixed and stained with a Rep-specific antibody. Surprisingly, CLSM revealed that unlike Rep-K340H, Rep-D371Y supported the formation of pAV2-LacO RCs (Fig 2E). A cell expressing wt Rep is shown as a positive control and a cell expressing mRFP was used as a negative control (Fig 2E).

The capability of Rep-D371Y to support AAV2 replication was also examined by Southern analysis. For this, Vero cells were co-transfected with the AAV2 replicon pAV2GFP and individual Rep constructs. The pAV2GFP replicon contains a recombinant AAV2 genome in which a green-fluorescent protein (GFP) transgene cassette is flanked by the AAV2 ITRs. At 24 hrs after transfection, the cells were infected with wt HSV-1 (MOI of 2) to provide helper factors for AAV2 replication. Three days after infection, extra-chromosomal DNA (Hirt DNA) was extracted, digested with DpnI to eliminate bacterial input DNA and subjected to Southern analysis using a GFP-specific DNA probe. As shown in Fig 2F, monomeric (ITRm) and dimeric (ITRd) double-stranded (DpnI resistant) as well as ITR ssDNA replication products of the recombinant AAV2 genome were detected in presence of both wt Rep and the mutant Rep-D371Y, but not in presence of the helicase null mutant Rep-K340H.

### The mutant Rep-D371Y proteins are less cytotoxic than the wt Rep proteins

**Fig 3:**
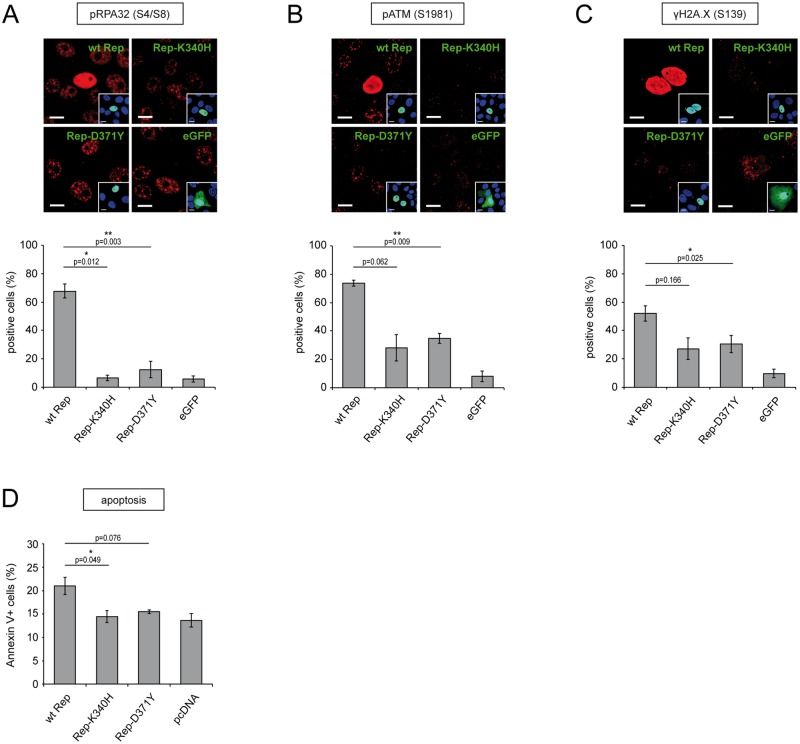
Impact of the different Rep constructs on the induction of a Rep-specific DNA damage response and apoptosis. (A-C) Assessment of Rep induced DNA-damage responses. Vero cells were transfected with plasmids encoding wt Rep, Rep-K340H, Rep-D371Y or eGFP as a negative control. Two days later, the cells were fixed and stained with antibodies specific for Rep (green; insets) and (red) either pRPA32-S4/8 (A), pATM-S1981 (B) or γH2A.X-S139 (C). The cell nuclei were visualized by staining with DAPI (blue; insets). Rep^+^- or GFP^+^-cells were scored for staining of the DDR markers using a confocal laser scanning microscope (graphs in A-C). Bars represent mean values (% positive cells) and SEs from 3 individual experiments. Asterisks indicate statistically significant differences between the positive control (wt Rep) and the corresponding mutant Rep constructs in a paired two-tail Student t-test (*, P<0.05; **, P<0.01). Scale bars, 10μm. (D) Screening of the Rep constructs for their ability to induce apoptosis in transfected cells. Vero cells were co-transfected with an eGFP expressing plasmid and a plasmid encoding either wt Rep, Rep-K340H, Rep-D371Y or no Rep (pcDNA). Three days later, the cells were stained with Cy5-conjugated annexin V and analyzed by flow cytometry with filters specific for eGFP (transfected cells) and Cy5 (apoptotic cells). The data are shown as means ± SE from three independent experiments (*, P<0.05).

In previous studies we and others reported that the AAV2 Rep68 and Rep78 proteins induce a distinct DNA-damage response (DDR) in transfected cells. This DDR is characterized by the activation (i.e. phosphorylation) of the replication protein A32 (RPA32), the sensor-kinase ataxia-telangiectasia mutated (ATM) and the histone 2A.X (H2A.X) [34, 36, 38, 65]. In particular, we found that the Rep domains necessary to induce the cellular DDR are the combined DNA-binding and ATPase/helicase domains [38]. Here, we assessed to what extent the mutant Rep-D371Y protein is capable of inducing a Rep-specific DDR in transfected cells. For this, Vero cells were transfected with the different Rep constructs (wt Rep, Rep-K340H, Rep-D371Y) or a plasmid expressing the gene for enhanced green-fluorescent protein (eGFP). The cells were fixed 48 hrs post transfection and stained with antibodies specific for either pRPA32 (S4/S8), pATM (S1981) or γH2A.X (S139). Expression of the different Rep constructs was confirmed with antibodies specific for Rep (Fig 3A–3C). Cells positive for Rep or eGFP were counted and scored for the staining of the different DDR markers (Fig 3A–3C). Interestingly, the number of cells displaying a DDR characterized by the different DDR markers was significantly smaller (20–60% reduction) upon transfection of the Rep-D371Y encoding plasmid than upon transfection of the wt Rep encoding plasmid. Similar to a previous study [38], the DDR induced by Rep-K340H was also significantly reduced (85–95% reduction) compared to the positive control (wt Rep) and was comparable to that induced by the mutant Rep-D371Y (Fig 3A–3C). However, it is important to note that the DDR characterized by pATM (S1981) or γH2A.X (S139) was not fully abolished in cells expressing the mutants Rep-K340H or Rep-D371Y when compared to the negative control (no Rep, eGFP). Interestingly, this is in accordance with the fact that pHSVGFP vector titers were also not fully restored in cells expressing these mutant Rep proteins (Fig 2C).

In a next step, we examined the capability of Rep-D371Y to induce apoptosis in transfected cells, since it has been shown that the Rep domains involved in DDR induction coincide with the domains involved in the induction of apoptosis [38, 66]. For this, Vero cells were co-transfected with the different Rep constructs together with a plasmid expressing eGFP to identify successfully transfected cells. Three days later, the cells were stained with Cy5-conjugated annexin V and subjected to flow cytometry. In particular, eGFP-positive cells were screened for staining of Cy5 as a marker for the induction of apoptosis (Fig 3D). Similar to the mutant Rep-K340H, the mutant Rep-D371Y did not lead to an increased number of Cy5-positive cells compared to the control (no Rep, pcDNA) while the number of apoptotic cells expressing wt Rep was significantly higher. Taken together, these results indicate that in transfected cells the overall cytotoxicity of the mutant Rep-D371Y proteins is clearly reduced compared to the wt Rep proteins.

### Production of recombinant AAV2-D371Y results in poor virus titers and disproportional accumulation of empty particles

**Fig 4:**
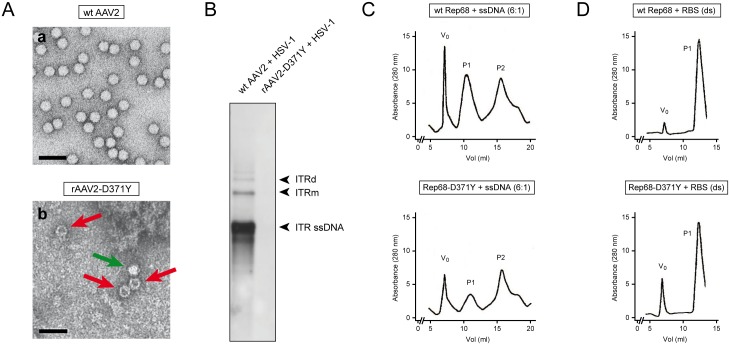
The mutant rep-D371Y gene is not compatible with the production of recombinant AAV2 virus stocks. (A) Wild-type (wt AAV2, a) and recombinant (rAAV2-D371Y, b) virus stocks were visualized and analyzed with transmission electron microscopy. Red arrows: empty particles (electron dense), green arrow: fully packaged particle (electron light). Scale bars; 50nm. (B) Southern analysis to assess replication of wt and recombinant AAV2 virus stocks. Vero 2–2 cells were co-infected with either wt (wt AAV2) or recombinant (rAAV2-D371Y) (MOI 20) and HSV-1 (MOI 1) followed Hirt DNA extraction 48hrs later. Southern blotting was performed as described in Fig 2F with a DIG-labeled probe specific for Rep. The ITR ssDNA, the monomeric (ITRm) and the dimeric (ITRd) AAV2 replication intermediates are indicated on the right. (C, D) The capability of the mutant Rep68-D371Y proteins to bind ssDNA is reduced, whereas specific binding of dsDNA is not affected. Gel filtration chromatography profiles of either wt Rep68 or mutant Rep68-D371Y proteins were utilized to assess the binding capacity to (C) unspecific ssDNA or (D) specific dsDNA (RBS) templates. V_0_ is the void volume where aggregates are eluted with a molecular mass larger than the exclusion limit of the column (indicating complex Rep multimers). The other species at P1 represent the Rep-dsDNA or Rep-ssDNA complexes respectively. The P2 species represent unbound Rep proteins.

To further study the impact of the mutant Rep-D371Y proteins on the AAV2 life-cycle, we sought to produce recombinant (r)AAV2-D371Y virus stocks. Production and purification of rAAV2-D371Y stocks was performed as described previously [52]. Surprisingly, rAAV2-D371Y virus stocks suffered from poor genomic virus titers (100-fold reduced compared to wt AAV2 virus stocks (data not shown)). Furthermore, electron micrographs of wt AAV2 and rAAV2-D371Y virus preparations revealed disproportional accumulation of unpackaged virus particles in the mutant rAAV2-D371Y virus stock whereas wt AAV2 stocks contained negligent numbers of empty capsid particles (Fig 4A). Moreover, co-infection of recombinant AAV2-D371Y and wt HSV-1 did not result in successful AAV2 DNA replication as shown with Southern analysis using a Rep-specific probe to detect AAV2 DNA replication intermediates (Fig 4B). In contrast, co-infection of wt AAV2 and HSV-1 resulted in the accumulation of the expected AAV2 replication intermediates ITR ssDNA, ITRm and ITRd. These findings indicate that the mutant Rep-D371Y proteins may be deficient for packaging of the viral ssDNA genome into pre-assembled virus capsids which results in rAAV2-D371Y stocks that are not compatible with efficient virus replication. This could be due to two reasons: first, the mutant Rep proteins may not be capable of binding to pre-assembled capsids, a process necessary to form the ssDNA-capsid complex [32, 67], or second, the mutant Rep proteins are not capable of binding to ssDNA. To test the latter possibility, we performed gel filtration chromatography to assess the capability of Rep-D371Y to form DNA-protein complexes with either ssDNA or dsDNA. To do so, we incubated purified wt Rep68 or mutant Rep68-D371Y proteins with either random ssDNA or RBS specific dsDNA substrates and assessed the formation of protein-DNA complexes by gel filtration followed by protein chromatography. Indeed, the capability of mutant Rep68-D371Y proteins to bind random ssDNA is substantially reduced compared to wt Rep68 proteins (Fig 4C). Interestingly, binding to ssDNA substrates seems to be not completely abolished as the peak of the P1 species, showing the mutant Rep68-D371Y-DNA complex, is not fully reduced as expected. However, dsDNA binding capacity was not affected by the mutant Rep68-D371Y proteins (Fig 4D).

### Incorporating the mutant *rep-D371Y* gene into HSV/AAV hybrid vectors significantly increases vector titers

**Fig 5:**
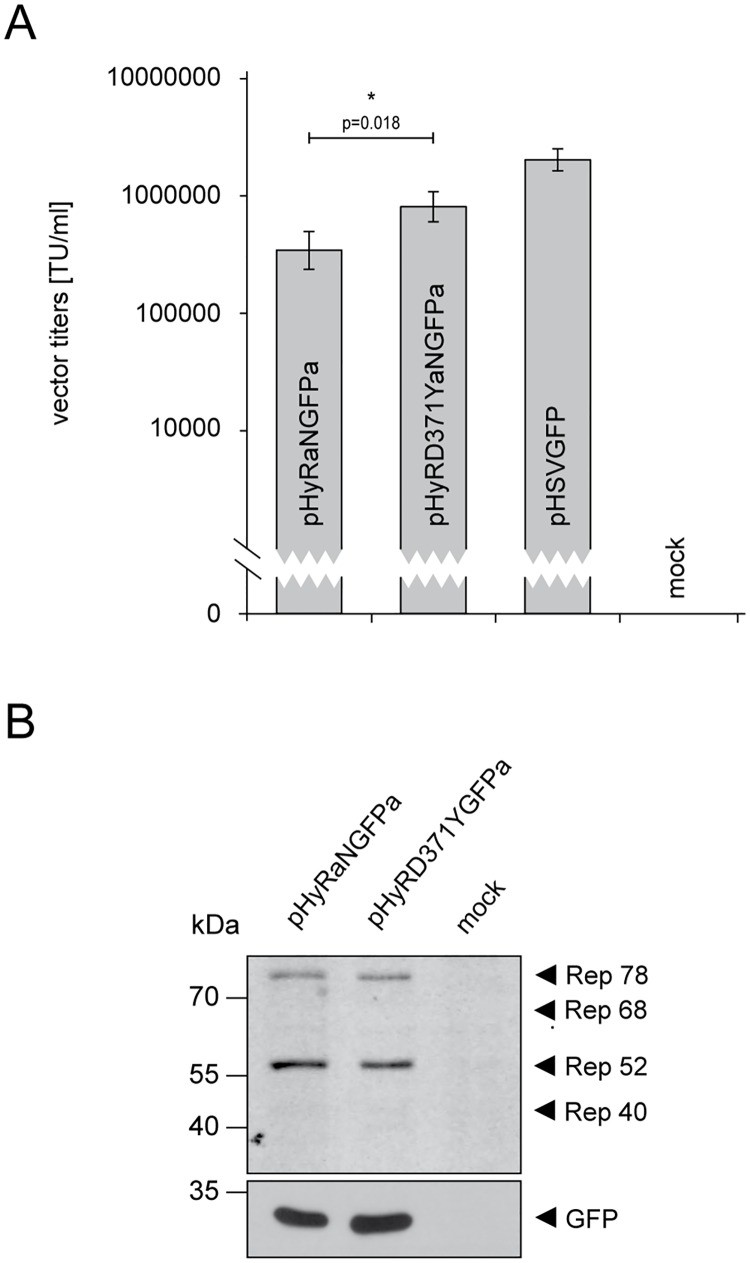
Virus titers of HSV/AAV hybrid vectors harboring the mutant *rep68/78-D371Y* gene are significantly higher than titers of hybrid vectors harboring the wt *rep68/78* gene. (A) Packaging and titration of the HSV/AAV hybrid vectors pHyRaNGFPa and pHyRD371YaNGFPa was performed as described for the HSV-1 vector pHSVGFP (Fig 2C). The data are shown as means ± SE from three independent experiments (*, P<0.05). (B) Western analysis to confirm Rep expression in HSV/AAV hybrid vector producing Vero-2-2 cells. Production of vectors was performed as described for Fig 2C and Fig 5A. Instead of harvesting vector particles, the cells were processed for and subjected to Western analysis. In addition to the Rep-specific antibody to detect Rep expression, the blots were stripped and re-stained with an antibody specific for GFP as a loading and expression control.

Due to the fact that the Rep-D371Y proteins are less cytotoxic than the wt Rep counterparts, this mutant Rep variant may be an interesting tool for vector development. For example, HSV/AAV hybrid gene transfer vectors have previously been developed to combine the advantageous properties of AAV2 (persistent transgene expression) and HSV-1 amplicon vectors (large transgene capacity [53, 68, 69]. HSV-1 amplicon vectors are bacterial plasmids that contain a transgene cassette and two *cis* elements from the HSV-1 genome, in particular an origin of DNA replication (*ori*) and a DNA packaging/cleavage signal (*pac*), which allow the replication and packaging of the double-stranded amplicon DNA into HSV-1 particles in presence of HSV-1 helper factors [58]. In addition to the HSV-1 *ori* and *pac* signals, HSV/AAV hybrid vectors contain the AAV2 *rep* gene and a transgene cassette that is flanked by AAV2 ITRs. The presence of these AAV2 elements supports long-term transgene expression and Rep-dependent integration of the ITR-flanked transgene cassette into the host genome [53]. However, the presence of the AAV2 *rep* genes on the vector genome resulted in low hybrid vector titers ([53] and Fig 5A). Alternatively, the incorporation of the mutant *rep68/78-D371Y* gene in place of the wt *rep68/78* gene may overcome the poor performance of the first generation HSV/AAV hybrid vectors. Indeed, the titers of HSV/AAV hybrid vectors encoding the mutant Rep68/78-D371Y proteins (pHyRD371YaNGFPa) were 5 to 10-fold higher compared to hybrid vectors encoding the wt Rep68/78 proteins (pHyRaNGFPa) (Fig 5A). Rep protein expression during packaging of the hybrid vectors was readily observed by Western analysis and was at comparable levels (Fig 5B).

## Discussion

In this study, we show that a novel AAV2 Rep helicase mutant, Rep-D371Y, has the unexpected property that it can support AAV2 DNA replication without blocking HSV-1 DNA replication. Moreover, compared to the wt Rep, the capacity of Rep-D371Y to induce apoptosis and a Rep-specific DDR was significantly reduced and comparable to that of Rep-K340H, a helicase-null mutant that neither supports AAV2 replication, nor blocks HSV-1 DNA replication. In addition, we compared the capability of the wt Rep and mutant Rep-D371Y proteins to bind either ssDNA or RBS dsDNA. The properties of the different wt and mutant AAV2 Rep proteins are summarized in Table 1.

**Table 1:** Summary of AAV2 Rep activities.

	AAV2 replication	HSV-1 replication	DNA damage response	Apoptosis	bind ssDNA	bind dsDNA
**wt Rep**	+++	-	+++	+++	+++	+++
**Rep-K340H**	-	++	+	-	n/a	n/a
**Rep-D371Y**	+++	++	+	-	+	+++

The activities of the wt Rep, the mutants Rep-K340H and Rep-D371Y were scored for their ability to (i) enable AAV2 replication, (ii) inhibit HSV-1 replication, (iii) induce a DNA damage response (DDR), (iv) induce apoptosis, (v), bind ssDNA and (vi) bind dsDNA; (-) no score, (+) minimal score, (++) intermediate score, (+++) high score.

The molecular interactions of the AAV2 Rep proteins with its helpervirus HSV-1 has been studied extensively in the past. We and others have demonstrated that the AAV2 Rep domains responsible for the inhibition of HSV-1 DNA replication include the combined DNA-binding and the ATPase/helicase domains [38, 41]. Direct binding of the AAV2 Rep proteins to the HSV-1 DNA and the subsequent activity of the ATPase/helicase domain have been shown to account at least in part for the Rep-mediated inhibition of HSV-1 replication [44]. This interaction may cause DNA lesions which are not compatible with effective HSV-1 DNA replication. However, the exact mechanism of interaction, in particular the impact of the Rep ATPase/helicase activity (i.e. unwinding) on the HSV-1 DNA itself remains elusive. Highly quantitative helicase assays are required to assess the Rep-specific unwinding process, which seems to be a hallmark of the Rep-mediated inhibition of HSV-1 replication. A key to understanding this mechanism may be the novel mutant Rep-D371Y. Evidentially, the findings presented here suggest that the mutant Rep-D371Y proteins allow the simultaneous and efficient replication of both HSV-1 and AAV2 DNA in the same cell. We indeed observed mature RCs of both viruses in the same cell when Rep-D371Y is present, while in presence of Rep-K340H only HSV-1 RCs but no AAV2 RCs are observed and in presence of wt Rep only AAV2 RCs but no HSV-1 RCs can be found respectively (data not shown and [37]).

The specific properties of the mutant Rep-D371Y protein make it an interesting tool for studying DDR in AAV2 and helpervirus co-infected cells. In particular, Rep-D371Y may allow dissecting the contributions of the different virus components to the DNA damage response observed in AAV2 and helpervirus co-infected cells. In particular, HSV-1 provokes a distinct DDR in infected cells, which is characterized by the activation of a cellular DNA double-strand break response pathway involving ATM, p53 and RPA [70–73], while the ATR response is inhibited [74]. Importantly, in HSV-1 infected cells, the catalytic subunit of the DNA-dependent protein kinase (DNA-PKcs) is degraded through ICP0-dependent proteasomal degradation [75, 76]. In HSV-1 and AAV2 co-infected cells, the degradation of DNA-PKcs is delayed in an AAV2-dependent manner, and DNA-PKcs in fact is recruited into AAV2 RCs [77, 78]. The modulation of the HSV-1 induced DDR by AAV2 may be due to the AAV2 genome (i.e. the single-stranded AAV2 DNA genome and its replication intermediates) and/or the AAV2 Rep proteins. The contribution of Rep could largely and specifically be eliminated by employing the mutant Rep-D371Y, while the contribution of the helpervirus could be eliminated largely by using an ICP0-deficient HSV-1 [77]. It will be particularly interesting to assess to what extent AAV2 DNA replication is affected by helpervirus-mediated and/or AAV2-mediated DNA damage responses and to study the dynamics and activation of specific DDR proteins such as RPA, ATM and γH2A.X in cells that support the simultaneous replication of both HSV-1 and AAV2.

Furthermore, it will be significant to study the performance of the mutant Rep-D371Y in specific steps of the AAV2 life-cycle such as (i) second-strand synthesis, (ii) genomic integration and (iii) genome packaging which all involve the Rep-helicase activity [31, 32]. Notably, we were not able to successfully produce recombinant AAV2 progeny virus stocks harboring the mutant *rep-D371Y* gene. Hence, we hypothesized that the mutant Rep-D371Y proteins may be deficient for packaging of the viral ssDNA genome into pre-assembled virus capsids, a process involving both, the binding of Rep to pre-assembled capsids and the formation of Rep-ssDNA complexes at the same time [32, 67]. A variety of mutations in the Rep SF3 helicase have been tested in the past for their ability to form the Rep-capsid complex. The three helicase mutants K391I, K391T and K404T showed reduced capsid complex formation and therefore reduced packaging activity, while the helicase mutants E379K, E379Q and K404I formed complexes as efficiently as wt Rep, but failed to package the AAV2 genome regardless [32]. This finding suggests that Rep-capsid complex formation is not affected when mutating the Glutamic-acid (E) at position 379, a residue very close and with similar biochemical properties to the Aspartic-acid (D) at position 371. Therefore, we concluded that Rep-capsid complex formation of the mutant Rep-D371Y may not be impaired either. However, the capability of mutant Rep68-D371Y proteins to bind ssDNA is substantially reduced compared to wt Rep68 proteins. Gel filtration chromatography assays revealed that mutant Rep68-D371Y proteins were not able to efficiently form protein-DNA complexes with ssDNA substrates, while the capacity of binding RBS dsDNA templates was not affected. This is in agreement with the fact that AAV2 Rep proteins facilitate specific binding of RBS dsDNA via their N-terminal DNA-binding domain, whereas binding of ssDNA is essentially mediated by the helicase domain which has no binding specificity. Notably, we exclude the possibility that the reason for unsuccessful production of a rAAV2-D371Y stock was due to the incompatibility of the mutant Rep-D371Y proteins to successfully replicate the viral genome. This is due to the fact that the AAV production system we utilized here is based on the replication of a dsDNA viral genome provided on a plasmid (pAV2 or pAV2-D371Y). Since we demonstrated that DNA replication from a viral dsDNA amplicon template is not affected by the mutant Rep-D371Y proteins (Fig 2F), we assumed that DNA replication may not be the limiting step during production of rAAV2-D371Y stocks.

Last but not least, we have demonstrated that the production efficiency of HSV/AAV hybrid vector stocks is significantly increased when we replace the wt *rep* gene with the mutant *rep-D371Y* gene in this vector. This effect may be due to the reduced capability of the mutant Rep-D371Y proteins to inhibit HSV-1 DNA replication. Intriguingly, Conway and colleagues developed an HSV-1 based AAV vector system, which was successfully utilized to produce recombinant AAV (rAAV) vectors in HEK293 cells [79]. In particular, rAAV vector stocks were produced by transfecting cells with a recombinant AAV plasmid followed by infection with an ICP27 deficient HSV-1 vector, which expresses the wt AAV Rep and Cap proteins (*d27*.*1-rc*). Unexpectedly, production of *d27*.*1-rc* was not affected by the presence of the wt *rep* gene. However, *d27*.*1-rc* was created by co-transfection of d27.1-lacZ infected cell DNA and a linearized integration plasmid harboring the *rep* and *cap* genes [79]. Since this type of HSV-1 vector production does not directly rely on HSV-1 DNA replication, it therefore may not be affected by the Rep-mediated inhibition of HSV-1 replication, which particularly involves blocking of the HSV-1 DNA amplification [38, 44].

The findings we present here substantially add to our understanding how and also to what extent the AAV2 Rep-helicase mechanistically contributes to different steps of the AAV2 life-cycle. In particular, we provide evidence that the Rep-helicase functions responsible for the induction of a DDR or apoptosis may not be identical to those critical for AAV2 replication, but they clearly coincide with the ability to inhibit HSV-1 replication. In addition, the novel AAV2 mutant Rep-D371Y proteins may allow deciphering yet unsolved Rep activities, which contribute to several steps of the AAV2 life cycle, such as replication or genomic integration. Last, but not least, the novel mutant Rep-D371Y potentially is utilized for the development of safer and more reliable AAV2 gene therapy vectors.
